# Causal relationship between inflammatory proteins and attention deficit hyperactivity disorder: A serum-metabolites-mediated Mendelian randomization analysis

**DOI:** 10.1097/MD.0000000000048963

**Published:** 2026-05-29

**Authors:** Kangning Zhou, Qiang Zhang, Miaomiao Liu, Yurou Yan, Junhong Wang

**Affiliations:** a First Clinical School of Medicine, Beijing University of Chinese Medicine, Beijing, China; b Department of Pediatrics, Dongzhimen Hospital, Beijing University of Chinese Medicine, Beijing, China.

**Keywords:** attention deficit disorder with hyperactivity, causality, genome-wide association study, inflammation mediators, Mendelian randomization analysis, metabolomics

## Abstract

Neuroinflammation is a potential pathogenesis of attention deficit hyperactivity disorder (ADHD). However, its consistency and specific causality remain unclear. We investigated the causal relationship between 91 inflammatory proteins and ADHD and explored the potential mediating role of 1400 serum metabolites. We obtained genome-wide association study (GWAS) data for 91 inflammatory proteins (n = 14,824), 1400 metabolites (n = 8299) and ADHD (n = 38,691) from publicly available datasets. A bidirectional 2-sample Mendelian randomization (MR) analysis was conducted to assess causal relationships between the inflammatory proteins and ADHD. Subsequently, a 2-step MR-based mediation analysis was performed to evaluate whether identified inflammatory proteins influence ADHD via specific metabolites. Sensitivity analyses verified the robustness of the results. Genetically predicted higher CD40L receptor (CD40) levels were associated with a reduced risk of ADHD (IVW odds ratio: 0.931; 95% CI, 0.894–0.970; *P* = .001), whereas the other 90 circulating inflammatory proteins were not significantly correlated with ADHD after false discovery rate correction. No reverse causal associations were detected for ADHD on 91 circulating inflammatory proteins. The 2-step MR analysis suggested that CD40 levels may exert an indirect effect on ADHD via *N*-acetylneuraminate, although the mediation effect did not reach statistical significance (mediation effect: 0.005; 95% CI, −0.000 to 0.011; mediation proportion: −7.290%; 95% CI, −15.100% to 0.506%). No significant pleiotropy, heterogeneity, or outliers were identified. This study provides genetic evidence supporting a protective role of CD40 in ADHD and highlights a putative immunometabolic pathway linking CD40 to neurodevelopmental outcomes. Our findings demonstrate the utility of integrating proteomic and metabolomic data within an MR framework to prioritize causal intermediates, informing potential directions for biomarker discovery and targeted intervention in ADHD.

## Graphical abstract

**Figure:**
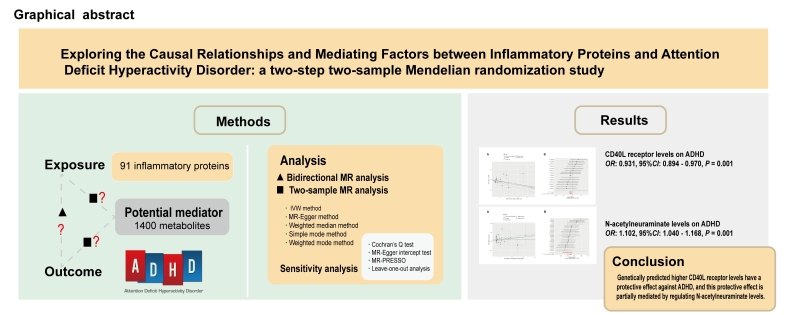

## 1. Introduction

Attention deficit hyperactivity disorder (ADHD) is a neurodevelopmental disorder that typically begins in childhood and is characterized by age-inappropriate hyperactivity, impulsivity, and/or inattention, leading to impaired social, academic, and family functioning.^[1]^ ADHD has an early onset and frequently persists into adulthood, with a prevalence of approximately 7.2% in children and 2.5 to 3.1% in adults.^[2,3]^ ADHD is also associated with an increased risk of psychiatric comorbidities and functional impairments across the lifespan.^[4]^ Although the etiology of ADHD remains unclear, increasing evidence suggests that neuroinflammation may contribute to its pathogenesis.^[5]^

Neuroinflammation, an immune response in the central nervous system triggered by disrupted homeostasis, involves activation of glial cells that release pro-inflammatory mediators and impair synaptic function and cognitive processes.^[6]^ Accumulating evidence has linked inflammatory pathways to ADHD. Genetic and proteomic studies have identified inflammation-related pathways and cytokines associated with ADHD.^[7-9]^ Experimental studies have demonstrated that neuroinflammatory activation increases inflammatory mediators such as tumor necrosis factor α (TNF-α), potentially affecting neuronal function.^[10]^ Clinical and epidemiological studies further indicate that immune-related disorders and maternal inflammatory conditions increase the risk of ADHD in offspring.^[11,12]^ Additionally, interventions targeting neuroinflammation have been reported to alleviate ADHD symptoms.^[13]^ However, most existing studies are observational or experimental, and systematic causal evidence remains limited.

Mendelian randomization (MR) uses genetic variants as instrumental variables to infer causal relationships between exposures and outcomes, thereby minimizing confounding and reverse causation.^[14]^ Given the growing but inconclusive evidence linking inflammation to ADHD, MR provides a robust approach to clarify potential causal associations. In this study, we conducted a bidirectional MR analysis using the latest genome-wide association study data of 91 inflammatory proteins to systematically evaluate their causal relationships with ADHD. Furthermore, a 2-step MR analysis was performed to explore potential metabolite-mediated pathways linking inflammatory proteins and ADHD. By elucidating inflammatory pathways and mediating mechanisms involved in ADHD, this study aims to provide deeper insights into disease pathogenesis and identify potential biomarkers and therapeutic targets.

## 2. Materials and methods

### 2.1. Study overview

**Figure 1.**
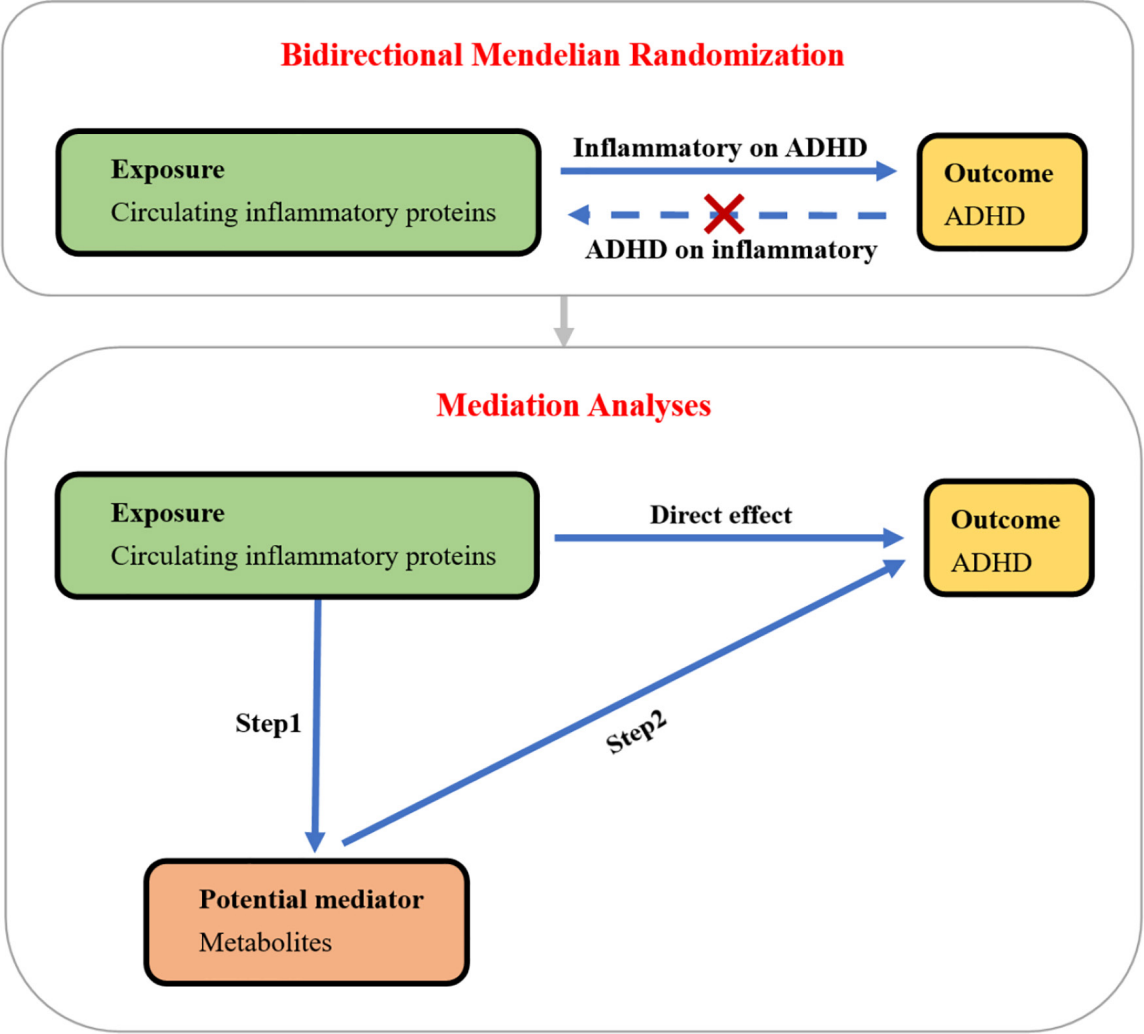
Study design of the Mendelian randomization analysis. ADHD = attention deficit hyperactivity disorder.

In the study, we obtained 91 circulating inflammatory proteins, ADHD and 1400 metabolites for a GWAS from publicly available datasets.^[15-17]^ We first investigated the causal relationship between exposure (91 circulating inflammatory proteins) and outcome (ADHD) through a 2-sample bidirectional MR study, using single-nucleotide-polymorphisms (SNPs) of 91 circulating inflammatory proteins and ADHD as instrumental variables (IVs), respectively. Therefore, we identified inflammatory proteins that had an adverse effect on ADHD and no causal relationship with ADHD in the opposite direction. Then, we used a 2-step MR to test the potential mediating effect of metabolites on the relationship between the identified circulating inflammatory proteins and ADHD. We the first step assessed the causal effects of these proteins on potential metabolites, and the second step focused on the effects of potential metabolites on ADHD by 2-sample MR. Finally, we performed a Mendelian mediation analysis to investigate the extent to which metabolites mediate the effect of inflammatory proteins on ADHD. Our study design is summarized in Figure 1.

### 2.2. Data sources

#### 2.2.1. Exposure: circulating inflammatory proteins

Genetic data for 91 circulating inflammatory proteins are from a meta-analysis that included 11 cohorts totaling 14,824 European participants.^[15]^ This study performed an extensive protein quantitative trait locus (pQTL) GWAS of 91 circulating inflammation-related proteins and quantified them by Olink immunoassay, identifying 180 major pQTL signals, of which 59 were cis signals and 121 were trans signals. For an in-depth look at the genetic and plasma proteomic data, please see the original publication.^[15]^ Detailed GWAS summary statistics for each protein were obtained from publicly available data in the EBI GWAS Catalog^[18]^ (accession numbers GCST90274758 to GCST90274848). The original studies obtained ethical approval and informed consent.

#### 2.2.2. Potential mediator: metabolites

GWAS data on metabolites were derived from a large GWAS of 8299 unrelated European individuals from the Canadian Longitudinal Study on Aging cohort.^[17]^ This GWAS included 1091 blood metabolites and 309 metabolite ratios, and summary statistics for all 1400 metabolites were obtained from publicly available data in the EBI GWAS Catalog^[18]^ (accession numbers GCST90199621 to GCST90201020).

#### 2.2.3. Outcome: ADHD

ADHD data were sourced from a GWAS meta-analysis by the Psychiatric Genomics Consortium.^[16]^ These data are from 38,691 ADHD patients and 1,86,843 controls, including Danish Integrative Psychiatric Research cohort, the Icelandic deCODE cohort and 10 European cohorts aggregated by the Psychiatric Genomics Consortium. The ADHD cases in these cohort studies met the ICD10 diagnosis of ADHD (F90.0, F90.1, F90.8) or took medication targeting ADHD symptoms, and the controls were individuals without ADHD. Informed consent was obtained from participants for all cohorts.

Population between exposure, potential metabolites and outcome groups will not overlap in the study. Details of the GWASs included in our study are provided in Tables S1 and S2, Supplemental Digital Content.

### 2.3. Selection of instrumental variable

MR design depends on 3 key assumptions for screening IVs: IVs are strongly associated with the exposure factors, there is no correlation between IVs and any potential confounding factors, IVs can only affect outcomes through exposure factors, not themselves or confounding factors.^[19]^

To ensure a sufficient number of independent IVs with strong statistical power, we selected SNPs at the genome-wide significance (*P* < 5 × 10^−5^) to ensure strong association with exposure and eliminated linkage disequilibrium using strict thresholds (*R*^2^ < 0.001 and kb > 10,000) to obtain independent IVs.^[20]^ Next, we addressed potential confounding utilizing the NIH LDlink LDtrait tool (https://ldlink.nih.gov/?tab=ldtrait) and identified potentially confounding phenotypes (e.g., phenotypes associated with the outcome and confounders. *R*^2^ ≥ 0.8, no proxies, and *P* < .001). SNPs associated with these confounding phenotypes were subsequently excluded. Finally, to adhere to the first assumption of MR, we assessed the strength of our IVs. We employed *R*^2^ as a genetic tool to estimate the proportion of phenotypic variance explained by each SNP (higher *R*^2^ indicates stronger individual SNP effects).^[21]^ Additionally, we selected SNPs with *F*-statistics > 10 to ensure all IVs have strong explanatory power.^[22]^

### 2.4. Statistical analyses

#### 2.4.1. Bidirectional 2-sample mendelian randomization

Five Mendelian randomization (MR) methods, including inverse variance weighted (IVW), MR-Egger, weighted median, simple mode, and weighted mode, were applied to investigate the causal relationship between circulating inflammatory proteins and ADHD.^[23-26]^ The IVW method was considered the primary analytical approach. This method assumes that all instrumental variables (IVs) are valid and free of horizontal pleiotropy and provides the most precise estimates based on multiplicative random-effects models. The causal estimates were presented as odds ratios (OR) with 95% confidence intervals (CIs). The IVW results were regarded as the main findings, whereas the other 4 MR methods were used as complementary analyses to evaluate the robustness and consistency of the results.^[27]^ Nominal significance was defined as a *P*-value < .05. Considering the multiple comparisons across the circulating inflammatory proteins, false discovery rate (FDR) correction using the Benjamini–Hochberg method was applied to adjust for multiple testing. An FDR-adjusted *q*-value < .05 was considered statistically significant.^[28]^

#### 2.4.2. Mediation analyses

A 2-step MR mediation analysis was conducted to investigate whether the identified circulating inflammatory proteins influenced ADHD through specific metabolites. In the first step, a 2-sample MR analysis was performed to evaluate the causal effect of circulating inflammatory proteins on metabolites. In the second step, a 2-sample MR analysis was applied to assess the causal effect of the identified metabolites on ADHD. Nominal significance was defined as a *P*-value < .05 in the IVW analysis. Subsequently, the product of coefficients method was used to estimate the mediation effect (indirect effect) of each metabolite.^[29]^ The indirect effect was calculated by multiplying the causal effect of circulating inflammatory proteins on metabolites by the causal effect of metabolites on ADHD. The proportion of the mediation effect was calculated by dividing the indirect effect by the total effect of circulating inflammatory proteins on ADHD.

#### 2.4.3. Sensitivity analyses

Sensitivity analyses were conducted to evaluate the robustness and reliability of the causal estimates.^[30]^ Horizontal pleiotropy was assessed using the MR-Egger intercept test, where an intercept closer to 0 indicates less pleiotropy.^[27,31]^ The Mendelian randomization pleiotropy residual sum and outlier (MR-PRESSO) method was used to detect potential pleiotropy and outlier SNPs (*P* < .05), and to correct causal estimates after removing outliers.^[32]^ Heterogeneity among IVs was evaluated using Cochran’s *Q* test, with *P* < .05 indicating significant heterogeneity.^[33]^ In addition, a leave-one-out analysis was performed to assess whether any single SNP disproportionately influenced the causal association.

### 2.5. Analysis tools

All statistical analyses were performed using R software (version 4.3.0; R Foundation for Statistical Computing) and RStudio (version 2023.06.2 Build 561; Posit Software, PBC). The following R packages were used: TwoSampleMR (version 0.5.8), data.table (version 1.14.8), MR-PRESSO, and LdlinkR.

## 3. Results

### 3.1. Relationship between circulating inflammatory proteins and ADHD

**Figure 2.**
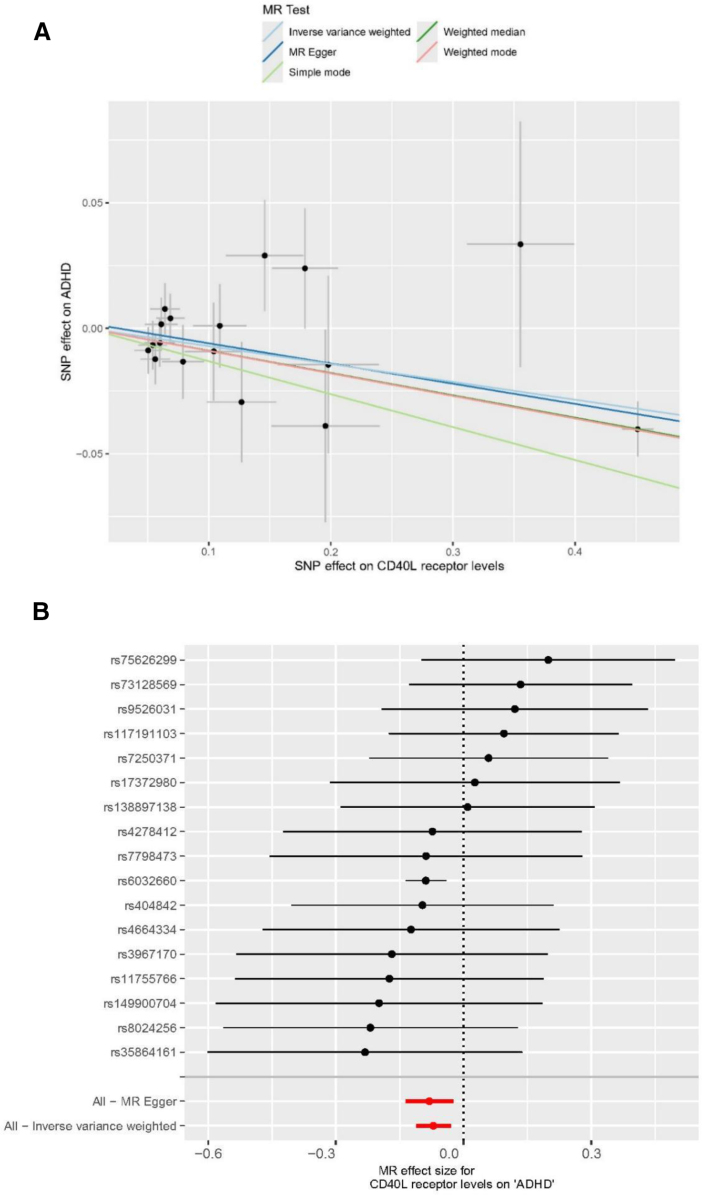
Mendelian randomization analysis of CD40L receptor levels and ADHD. (A) Scatter plot showing SNP effects on CD40L receptor levels versus ADHD, with fitted lines representing causal estimates from different MR methods. (B) Forest plot of individual SNP and combined MR effect estimates for CD40L receptor levels on ADHD. ADHD = attention deficit hyperactivity disorder, MR = Mendelian randomization, SNP = single-nucleotide-polymorphism.

The characteristics of SNPs used as IVs for the 91 circulating inflammatory proteins are detailed in Table S3, Supplemental Digital Content. The proportion of variance explained by all the variants (*R*^2^) that we used as IVs varied from 7.46% (for Interleukin-20 receptor subunit alpha levels) to 34.61% (for C – C motif chemokine 25 levels; Table S3, Supplemental Digital Content). The *F*-statistic of all these selected IVs were larger than 10 indicating our results were less likely to be biased by weak instrument. We conducted a 2-sample MR to investigate the impact of 91 circulating proteins on ADHD. Genetic estimates for the associations between these inflammatory proteins with ADHD are presented in Table S4, Supplemental Digital Content. The IVW analyses identified 5 circulating inflammatory proteins with nominally significant (*P* < .05) causal associations with ADHD. Eotaxin levels, glial cell line-derived neurotrophic factor, and leukemia inhibitory factor levels all showed positive association with ADHD (IVW OR, 1.081; 95% CI, 1.011–1.156; *P* = .022; IVW OR, 1.071; 95% CI, 1.000–1.147; *P* = .049), (IVW OR, 1.081; 95% CI, 1.011–1.156; *P* = .022; Table S5, Supplemental Digital Content). Conversely, urokinase-type plasminogen activator and CD40L receptor (CD40) levels were negatively associated with ADHD (IVW OR, 0.942; 95% CI, 0.892–0.994; *P* = .030), (IVW OR, 0.931; 95% CI, 0.894–0.970; *P* = .001; Table S5, Supplemental Digital Content). However, after False discovery rate (FDR) correction, only the genetic susceptibility of CD40 levels reached statistical significance (*q*- value = .005; Table 1). All SNPs served as IVs for CD40 levels, as well as the *F*-statistic and *R*^2^ of each SNP, are presented in Table S6, Supplemental Digital Content. Figure 2 shows scatter plots of CD40 levels related to ADHD, with colored lines representing the results of different MR analyses. Figure 2 also indicates forest plots visualizing the individual and combined SNP MR-estimated effect sizes. Leave-one-out analysis (Fig. S1, Supplemental Digital Content) revealed no single SNP dominated the MR results. Additionally, the symmetrical distribution of SNPs in the funnel plot (Fig. S1, Supplemental Digital Content) indicates a low likelihood of bias impacting the causal relationships. The associations of individual SNPs with CD40 levels and ADHD were presented in Table S7, Supplemental Digital Content. The MR-Egger intercept and MR-PRESSO analysis showed no evidence of horizontal pleiotropy (*P* > .05), and no outliers were detected by MR-PRESSO. The Cochran’s *Q*-test produced *P*-values above .05 for the remaining circulating inflammatory proteins, suggesting that no significant heterogeneity was found (Table 2).

**Table 1:** Significant Mendelian randomization results of circulating proteins on ADHD.

Method	nSNPs	*F*-statistic	*R* ^2^	β	OR (95% CI)	*P*-value	*q*-value
CD40L receptor levels							
IVW	17	99.666	10.861%	−0.071	0.931 (0.894–0.970)	.001	0.005
MR-Egger				−0.080	0.923 (0.872–0.976)	.013	
Weighted median				−0.089	0.915 (0.873–0.958)	<.001	
Simple mode				−0.131	0.877 (0.743–1.036)	.142	
Weighted mode				−0.089	0.914 (0.872–0.958)	.002	

*P*-values from the IVW MR test were adjusted using Benjamini–Hochberg FDR correction; for the resulting q-value threshold was set at <0.05.

CI = confidence interval, IVW = inverse variance–weighted, OR = odds ratio, SNP = single-nucleotide polymorphism.

**Table 2:** Pleiotropy and heterogeneity tests of instrumental variables for CD40L receptor levels in ADHD.

Exposure	nSNPs	Heterogeneity test				Pleiotropy test		
		IVW		MR-Egger		MR-Egger intercept	*P*	MR-PRESSO global test *P*
		Cochran’s Q	*p*	Cochran’s *Q*	*P*			
CD40L receptor levels	17	12.943	0.677	12.717	.624	0.002	.641	0.539

ADHD = attention deficit hyperactivity disorder, IVW = inverse variance weighted, MR = Mendelian randomization, MR-PRESSO = Mendelian randomization pleiotropy residual sum and outlier, SNP = single-nucleotide-polymorphism.

Using GWAS data on ADHD as exposure data, we performed MR analyses to investigate the causal effects of ADHD on 91 circulating inflammatory proteins. The SNPs served as IVs for ADHD are presented in Table S8, Supplemental Digital Content. Detailed information on the proportion of variance explained by the variants (*R*^2^) used as IVs is provided in Table S8, Supplemental Digital Content. Additionally, the *F*-statistic of all these selected IVs were larger than 10 indicating a lack of weak instrument bias (Table S8, Supplemental Digital Content). Our findings did not indicate a causal relationship for ADHD on the CD40 levels (IVW OR, 1.065; 95% CI, 0.979– 1.158; *P* = .143; Table 3, Fig. S2, Supplemental Digital Content). No pleiotropy, no heterogeneity, and no outliers were identified in our analyses (Table S9, Supplemental Digital Content).

**Table 3:** Bidirectional Mendelian randomization analysis between ADHD and CD40L receptor levels.

Method	nSNPs	*F*-statistic	*R* ^2^	β	OR (95% CI)	*P*-value
IVW	20	18.369	0.163%	0.063	1.065 (0.979–1.158)	.143
MR-Egger				-0.001	0.999 (0.564–1.771)	.998
Weighted median				0.056	1.057 (0.935–1.196)	.376
Simple mode				0.014	1.014 (0.791–1.300)	.912
Weighted mode				0.002	1.002 (0.815–1.232)	.988

ADHD = attention deficit hyperactivity disorder, CI = confidence interval, IVW = inverse variance weighted, OR = odds ratio, SNP = single-nucleotide-polymorphism.

### 3.2. Effect of circulating inflammatory proteins on metabolites

To further investigate the potential mediating pathway linking CD40 levels and ADHD, we conducted a 2-step MR analysis to determine if the impact of CD40 on ADHD is mediated by metabolites. Initially, we assessed the causal effects of CD40 levels on 1400 metabolites. We found that genetically predicted CD40 levels were associated with *N*-acetylneuraminate levels (IVW β, 0.053; 95% CI, 0.003 to 0.104; *P* = .037; Table 4, Fig. S3, Supplemental Digital Content). Summary information on *N*-acetylneuraminate levels for the SNPs associated with CD40 levels was listed in Table S10, Supplemental Digital Content. The associations of individual SNPs with CD40 levels and *N*-acetylneuraminate levels were presented in Table S11, Supplemental Digital Content. No pleiotropy, no heterogeneity, or outliers were observed in our analyses (Table S12, Supplemental Digital Content).

**Table 4:** Mendelian randomization analysis of CD40L receptor levels on *N*-acetylneuraminate levels.

Method	nSNPs	*F*-statistic	*R* ^2^	β (95% CI)	*P*-value
IVW	43	44.556	18.992%	0.053 (0.003–0.104)	.037
MR-Egger				0.074 (0.007–0.140)	.036
Weighted median				0.042 (−0.027–0.112)	.236
Simple mode				0.009 (−0.150–0.167)	.912
Weighted mode				0.041 (−0.031–0.112)	.271

CI = confidence interval, IVW = inverse variance weighted, SNP = single-nucleotide-polymorphism.

### 3.3. Effect of metabolites on ADHD

**Figure 3.**
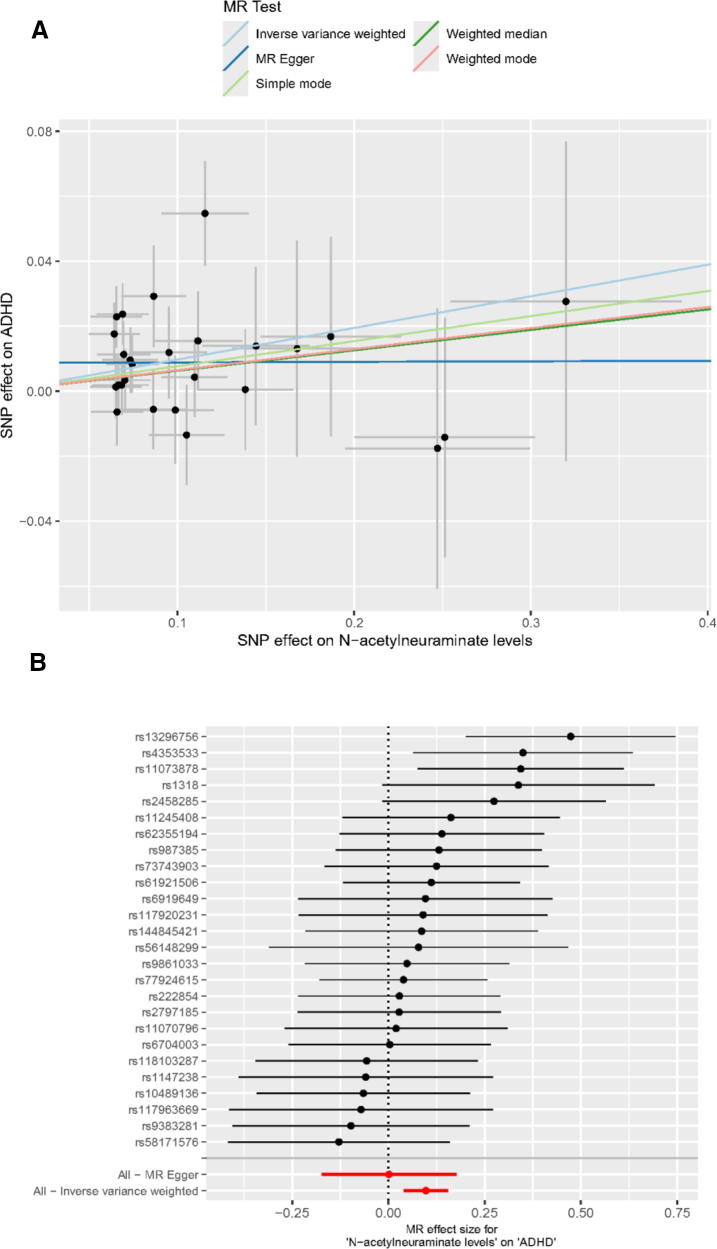
Mendelian randomization analysis of *N*-acetylneuraminate levels and ADHD. (A) Scatter plot showing SNP effects on *N*-acetylneuraminate levels versus ADHD, with fitted lines representing causal estimates from different MR methods. (B) Forest plot of individual SNP and combined MR effect estimates for N-acetylneuraminate levels on ADHD. ADHD = attention deficit hyperactivity disorder, MR = Mendelian randomization, SNP = single-nucleotide-polymorphism.

Subsequently, we evaluated the causal effect of *N*-acetylneuraminate levels on ADHD, utilizing genetic instruments linked to *N*-acetylneuraminate levels (Table S13, Supplemental Digital Content). Figure 3 and Table 5 shows the scatter plot and forest plot that our MR analyses supported that genetically predicted increased risk of ADHD was associated with genetically predicted *N*-acetylneuraminate levels (IVW OR, 1.102; 95% CI, 1.040–1.168; *P* = .001). The associations of individual SNPs with CD40 levels and *N*-acetylneuraminate levels were presented in Table S14, Supplemental Digital Content. No pleiotropy, no heterogeneity, and no outliers were identified in our analyses (Table S15 and Fig. S4, Supplemental Digital Content).

**Table 5:** Mendelian randomization analysis of *N*-acetylneuraminate levels on ADHD.

Method	nSNPs	*F*	*R* ^2^	β	OR (95% CI)	*P*-value
IVW	26	24.173	9.697%	0.097	1.102 (1.040–1.168)	.001
MR-Egger				0.002	1.002 (0.841–1.193)	.985
Weighted median				0.063	1.065 (0.987–1.149)	.107
Simple mode				0.077	1.080 (0.932–1.251)	.315
Weighted mode				0.065	1.067 (0.937–1.215)	.340

ADHD = attention deficit hyperactivity disorder, CI = confidence interval, IVW = inverse variance weighted, OR = odds ratio, SNP = single-nucleotide-polymorphism.

### 3.4. Mediation analyses

**Figure 4.**
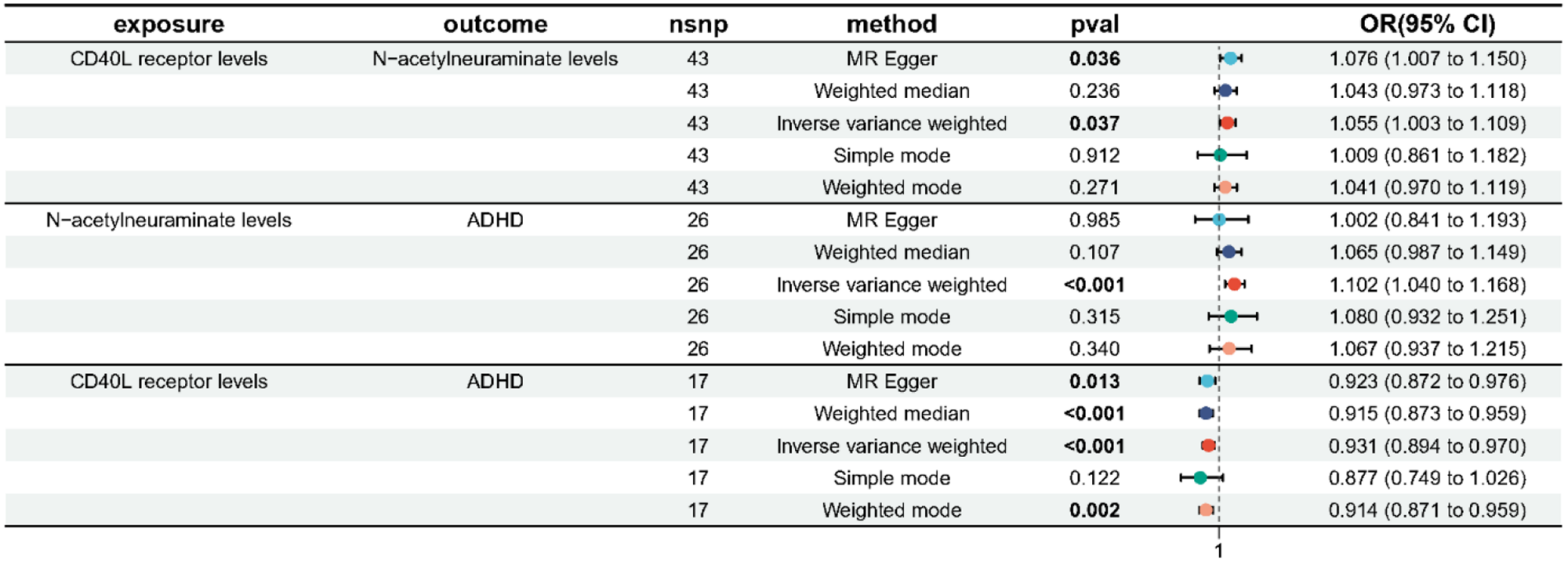
Overall forest plot of the Mendelian randomization results. ADHD = attention deficit hyperactivity disorder, CI = confidence interval, OR = odds ratio, SNP = single-nucleotide-polymorphism.

Given the observed causal association between *N*-acetylneuraminate levels and ADHD in MR analyses, we conducted mediation analyses to estimate the proportion of the effect of CD40 levels on ADHD mediated through *N*-acetylneuraminate. The results suggested that CD40 levels may influence ADHD risk indirectly via *N*-acetylneuraminate, although the mediation effect did not reach statistical significance (mediation effect: 0.005; 95% CI, −0.000 to 0.011; mediation proportion: −7.29%; 95% CI, −15.10% to 0.506%; Table 6). The overall forest plot is shown in Figure 4.

**Table 6:** Mediation analysis of CD40L receptor levels on ADHD via *N*-acetylneuraminate levels.

Exposure	Mediator	Outcome	Mediated effect	Mediated proportion	*P*-value
CD40L receptor levels	N-acetylneuraminate levels	ADHD	0.005 (−0.000 to 0.011)	−7.290% (− 15.100% to 0.506%)	.067

ADHD = attention deficit hyperactivity disorder.

## 4. Discussion

In this study, we used summary statistics from the largest GWAS meta-analysis of 91 circulating inflammatory proteins and ADHD to understand the causal relationship. By performing a bidirectional 2-sample MR analysis and FDR correction, we found causal evidence indicating a protective effect of the CD40 levels on ADHD. Interestingly, reverse MR indicates that ADHD itself did not seem to influence the levels of any of the 91 circulating inflammatory proteins. Furthermore, we conducted a mediation analysis and found that the protective effect of CD40 levels on ADHD was partially mediated by regulating *N*-acetylneuraminate levels. These findings could have implications for public health interventions aimed at reducing ADHD rates.

Inflammation is increasingly recognized as a key factor in many somatic pathologies, and strong evidence suggests that immune dysregulation and pro-inflammatory states are present in a variety of mental illnesses.^[34-37]^ However, evidence of neuroinflammatory processes in ADHD is relatively scarce compared to other mental illnesses. Multiple studies have investigated into the potential of neuroinflammation in ADHD, with some suggesting a link between peripheral inflammatory cytokines and the severity of ADHD symptoms.^[38,39]^ Higher levels of certain cytokines (IL-13, IL-16, IL-6, IL-10, IL-8, and TNF-α) are associated with an increased risk of attention issues in individuals with ADHD.^[38,40]^ A connection has also been established between ADHD and systemic autoimmune conditions like allergies and atopic diseases.^[41,42]^ While existing research suggests that neuroinflammation and immune dysregulation are potential contributing factors to ADHD, a definitive causal relationship between ADHD behaviors and inflammatory proteins has yet to be established.^[43]^ Additionally, despite advances in the field of psychiatry, inflammatory biomarkers are not routinely assessed in clinical practice, making MR an attractive approach.

As far as we know, this is the first study to explore the extent to which metabolites mediate a causal path between circulating inflammatory proteins and ADHD. We found the genetic liability for CD40 levels reached a statistical significance after FDR correction. CD40 and CD40L are members of the tumor necrosis factor superfamily (TNFRSF) and key regulators of the immune system, widely expressed in B cells, T cells, macrophages, and glial cells. Normally forward signaling by CD40L activates CD40 causing secretion of pro-inflammatory factors leading to inflammation. CD40, upon binding with CD40L, activates several signaling pathways, including phosphatidylinositol 3-kinase/Akt (PI3K), mitogen-activated protein kinase, and canonical and noncanonical NF-κB pathways. These pathways lead to the production of pro-inflammatory cytokines, cell adhesion molecules, and chemokines (IL-1, TNFα, IL-8, and vascular endothelial growth factor).^[44-46]^ Furthermore, bidirectional signaling by CD40L/CD40 plays an important role in regulating neuronal synaptic growth and remodeling during neural development.^[47,48]^ It has also been suggested that positive signaling by CD40L/CD40 can act as a growth promoter for axonal cells of neuronal cells independently of neurotrophins, and that CD40L/CD40 deficient mice neuronal cells have shorter axons. Also, the autocrine signaling loop of CD40L/CD40 affects neuronal axonal growth response to neurotrophins. Reverse signaling from CD40-activated CD40L promotes axon growth in excitatory neurons, with longer and more branched axons in wild-type mice compared to CD40-deficient mice and has the opposite effect on inhibitory neurons.^[47,49,50]^ This process involves protein kinase C (PKC), extracellular regulated kinases 1 and 2 (ERK1/2), and c-Jun N-terminal kinase (JNK) signaling networks.^[51,52]^ Our MR analysis revealed causal evidence suggesting that CD40 levels may have a protective effect against ADHD. Early life neuronal development is strongly associated with the development of ADHD.^[53]^ Unfortunately, there is only support in the literature for the involvement of CD40 in early neuronal development, and the exact relationship with ADHD remains to be further verified.

An intriguing finding from our research is that the second step analysis indicated a link between genetically elevated *N*-acetylneuraminate levels and an increased risk of ADHD. Our results suggested that the effect of CD40 levels on ADHD risk is partially mediated through increasing *N*-acetylneuraminate levels, with a mediation ratio of −7.29%. Metabolic processes of *N*-acetylneuraminate, also known as sialic acid (SA), are associated with neuronal growth, neuronal plasticity, and modulation of neural excitability, and process-related gene polymorphisms are associated with psychiatric disorders.^[54-56]^ It is well known that neuronal excitability regulation, neurological development, and the onset of ADHD are closely related.^[57-59]^ Previous studies have suggested that defects in gene St3gal3, a risk factor for ADHD, is an important gene involved in the metabolic pathway for SA.^[60]^ SA monomers can form polysialic acid (PSA), a significant component of neural cell adhesion molecules crucial for synaptic, neuronal functions, and memory formation.^[61-64]^ Some studies have reported that normal children have higher levels of SA than autistic children, and no correlation was found between SA levels and the severity of autism.^[65,66]^ It has been reported that a deficiency or severe reduction in SA can lead to developmental delays and death.^[67]^ However, our research suggests that SA is a risk factor for ADHD, which may seem contradictory to the conclusion that SA has neuroprotective properties. Conversely, some studies observed that there is a high level of PSA in schizophrenic patients and a high level of SA in autism spectrum disorders.^[68,69]^ Excessive sialylation of PSA in myelin sheaths can cause myelin sheath disintegration and reduced function, leading to decreased neuronal separation and sensitivity.^[70,71]^ Therefore, we can speculate that there is a delicate balance of SA in the body, with both deficiency and excess leading to abnormalities, making balance crucial.

Although there is currently no definitive evidence linking SA to various neurodevelopmental disorders, our study provides a foundation for further investigation into the relationship between SA and ADHD. SA remains a promising area of research for the prevention of brain-related diseases. Exploring the connection between SA and neurological disorders offers ample opportunities for developing therapeutic strategies based on SA metabolism.

Our study yielded intriguing conclusions: CD40 is a protective factor against ADHD, and an increase in CD40 levels elevates SA levels. However, SA is a risk factor for ADHD. This paradox may arise because we used only 1400 serum metabolites as intermediaries. CD40’s protective effect on ADHD might be mediated through other pathways. Additionally, SA metabolism involves complex pathways, and other mechanisms within these pathways might increase the risk of ADHD. These aspects warrant more in-depth research.

Our study has several strengths. As far as we know, this is the first study to explore the extent to which metabolites mediate a causal path between circulating inflammatory proteins and ADHD. We employed a MR design that leverages SNPs as IVs for CD40 levels. This approach mitigates the concerns of reverse causality and confounding, which are prevalent limitations of observational studies. All of our instrumental variable *F*-statistics were >10, suggesting that our study is not subject to weak instrument bias. Furthermore, the exposures and outcome were derived from the 2 largest GWAS conducted to date, utilizing data from 2 distinct, nonoverlapping populations. To minimize population stratification, we focused exclusively on the European population. Finally, the MR-Egger and MR-PRESSO analyses showed no presence of outliers, suggesting an absence of horizontal pleiotropy and strengthening the validity of our causal inferences.

Several limitations warrant consideration. First, the generalizability of our findings might be limited by the predominantly European ancestry of the GWAS data. Replication in more diverse populations is necessary. Second, our study likely overlooks additional mediators beyond those investigated. Further research is needed to explore this possibility. Third, ADHD has a male predominance, the potential for sex bias exists due to the unadjusted nature of our analyses for gender. Future studies should incorporate sex stratification to elucidate any sex-specific effects of inflammatory proteins on ADHD risk. Finally, the absence of publicly available stratified data hinders our ability to examine the influence of inflammatory proteins on ADHD progression, severity, and clinical subtypes. Future research efforts should prioritize the collection of such data.

## 5. Conclusion

In conclusion, this MR study provides genetic evidence suggesting a protective role of CD40 levels in ADHD. These findings suggest that CD40-mediated inflammatory signaling may represent a previously underrecognized pathway in ADHD etiology, offering a potential direction for the discovery of novel biomarkers or therapeutic targets.

Although our mediation analysis did not yield statistically significant evidence for N-acetylneuraminate as a definitive mediator, the observed indirect effect hints at a possible metabolic axis linking immune function to neurodevelopmental outcomes. Future studies are warranted to validate this putative pathway in independent cohorts and to explore its underlying biological mechanisms through functional experiments.

More broadly, this study demonstrates the utility of integrating proteomic and metabolomic data within a MR framework to prioritize causal intermediates. Expanding such integrative approaches to larger and more diverse populations will be critical for translating genetic findings into actionable insights for ADHD prevention and intervention.

## Acknowledgments

We are grateful for the Psychiatric Genomics Consortium (PGC) and the EBI GWAS Catalog for providing ADHD and 91 circulating inflammatory proteins and 1400 metabolites GWAS study summary data. We sincerely thank all investigators for sharing these data.

We also acknowledge the use of generative artificial intelligence tools (ChatGPT) to assist with language polishing and editorial refinement of the manuscript. All scientific content, data interpretation, and conclusions were independently reviewed and verified by the authors.

## Author contributions

**Conceptualization:** Kangning Zhou, Qiang Zhang, Miaomiao Liu, Yurou Yan.

**Data curation:** Kangning Zhou, Qiang Zhang.

**Formal analysis:** Kangning Zhou, Qiang Zhang.

**Funding acquisition:** Junhong Wang.

**Investigation:** Miaomiao Liu, Yurou Yan.

**Methodology:** Kangning Zhou, Qiang Zhang.

**Project administration:** Miaomiao Liu, Yurou Yan, Junhong Wang.

**Resources:** Kangning Zhou, Qiang Zhang, Miaomiao Liu.

**Software:** Kangning Zhou, Qiang Zhang.

**Supervision:** Junhong Wang.

**Validation:** Miaomiao Liu.

**Visualization:** Kangning Zhou, Yurou Yan.

**Writing – original draft:** Kangning Zhou, Qiang Zhang.

**Writing – review & editing:** Kangning Zhou, Qiang Zhang, Miaomiao Liu, Yurou Yan, Junhong Wang.






































