# Supplementary figures and images for: Causal relationship between inflammatory proteins and attention deficit hyperactivity disorder: A serum-metabolites-mediated Mendelian randomization analysis
Source: Medicine (Baltimore). 2026 May 29;105(22):e48963. doi: 10.1097/MD.0000000000048963 (PMC13225583; doi:10.1097/MD.0000000000048963)

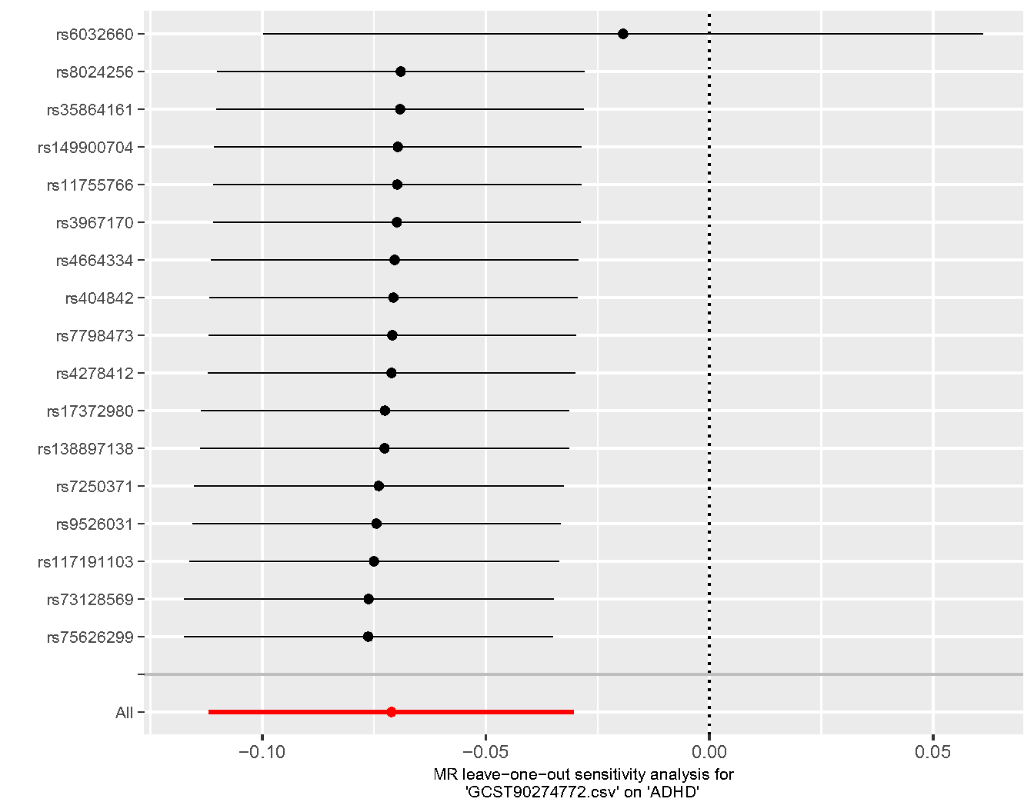


**Figure S1(A).**


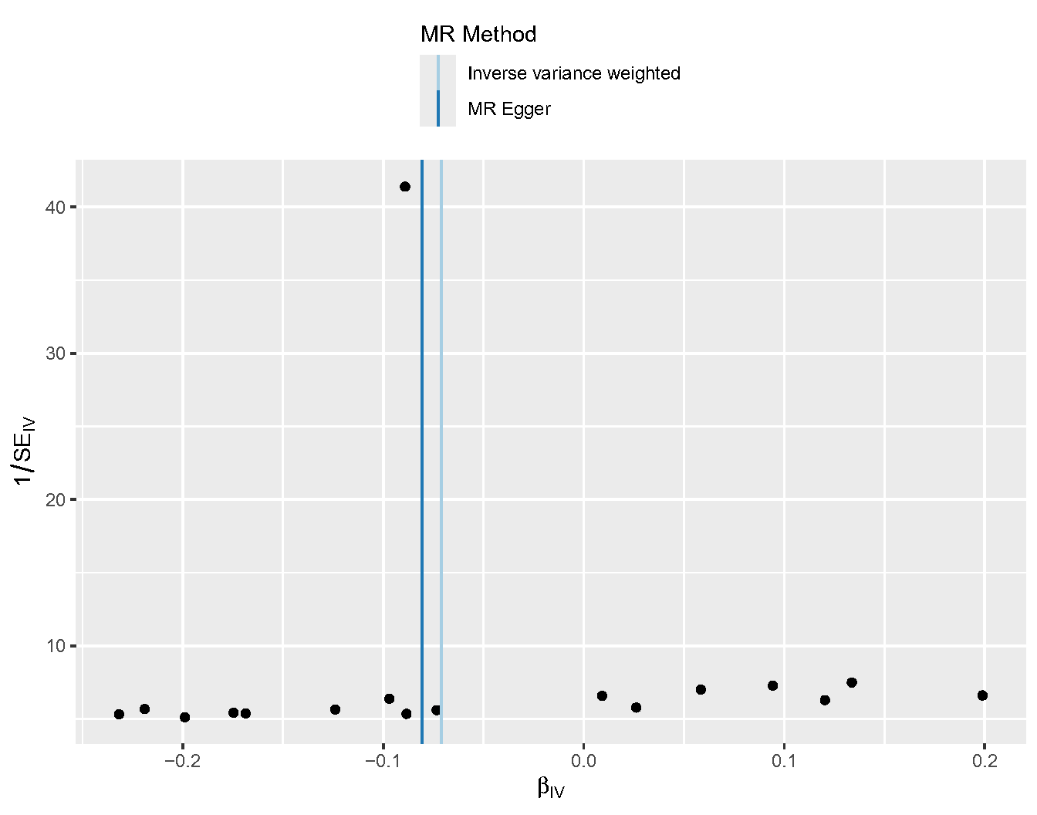


**Figure S1(B).**

Supplement: Supplementary file 6 [file medi-105-e48963-s006.docx]

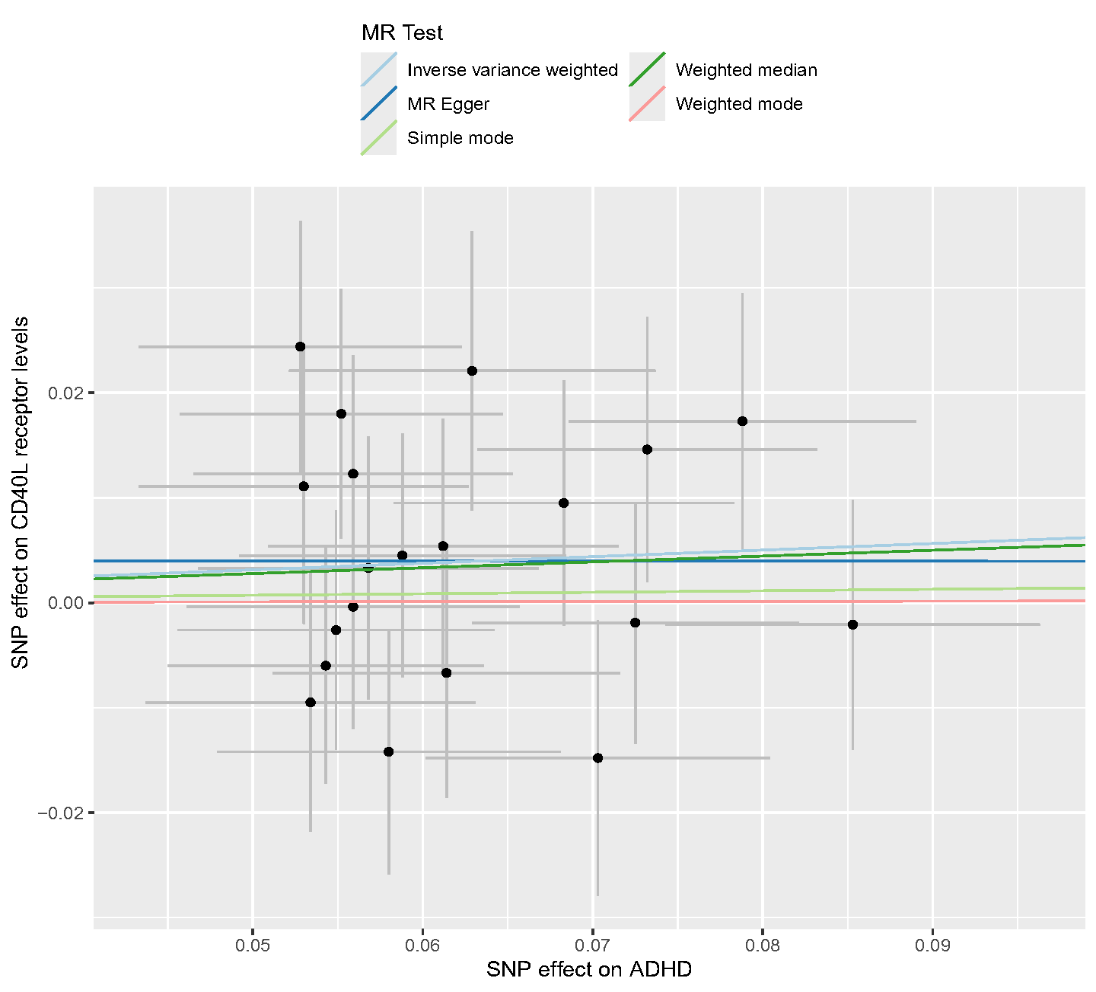


**Figure S2(A).**


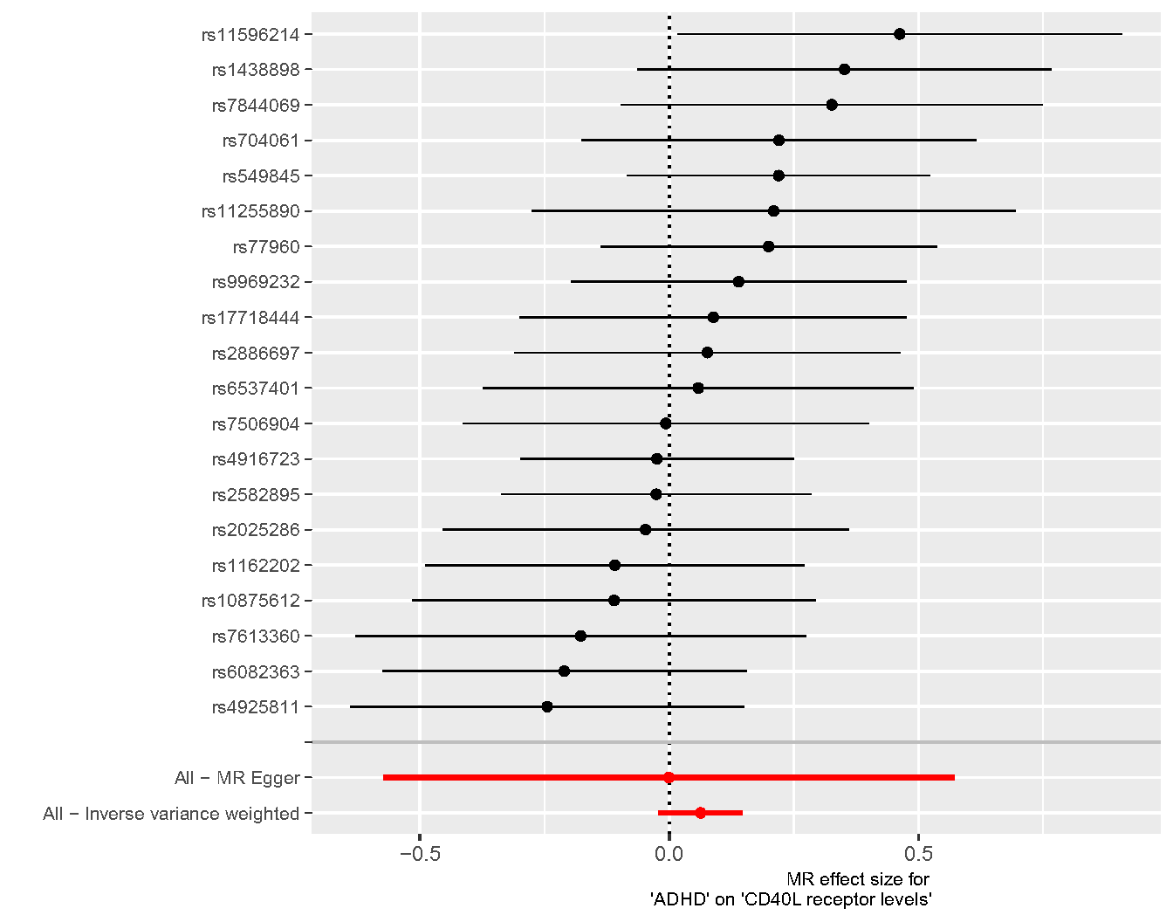


**Figure S2(B).**


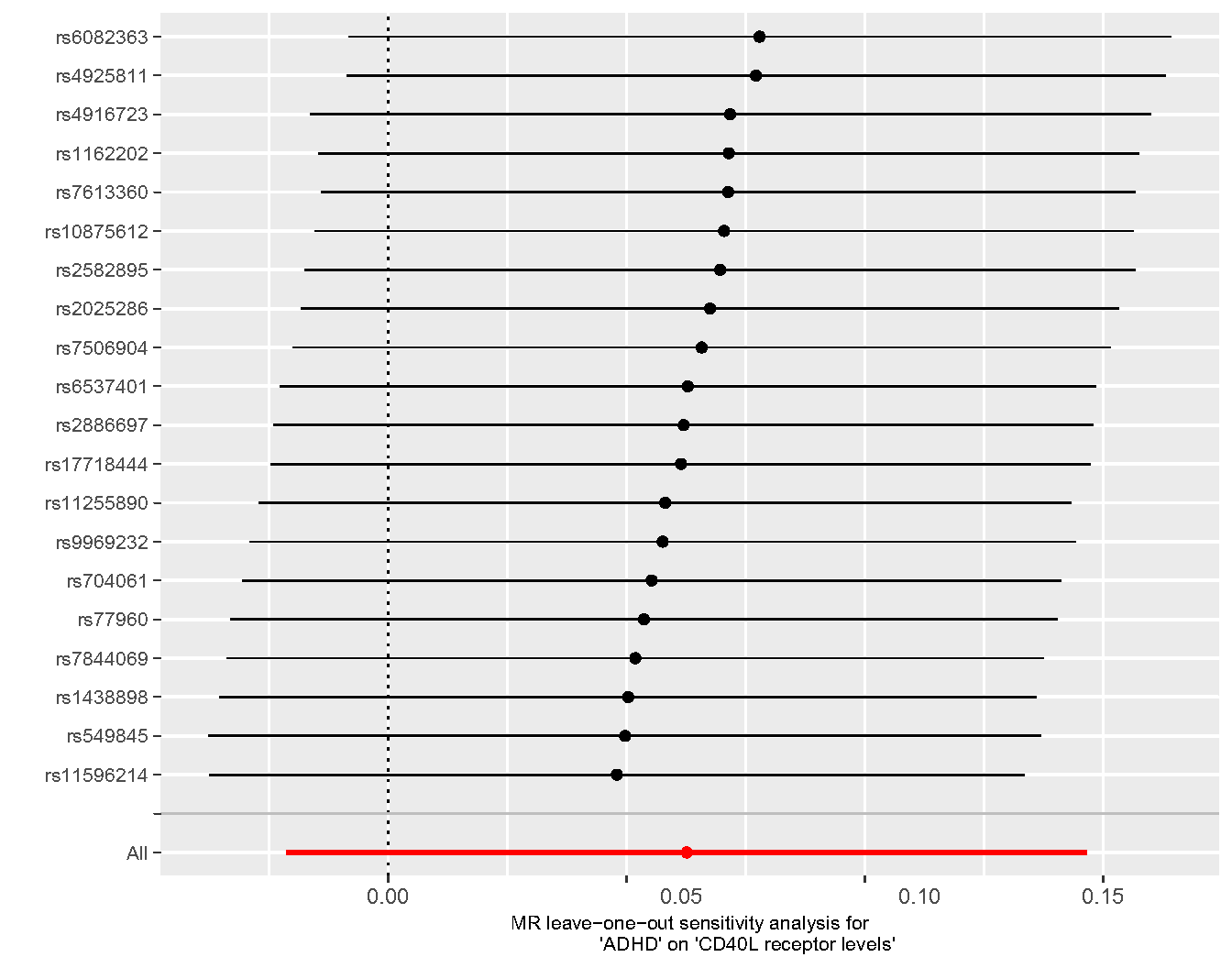


**Figure S2(C).**


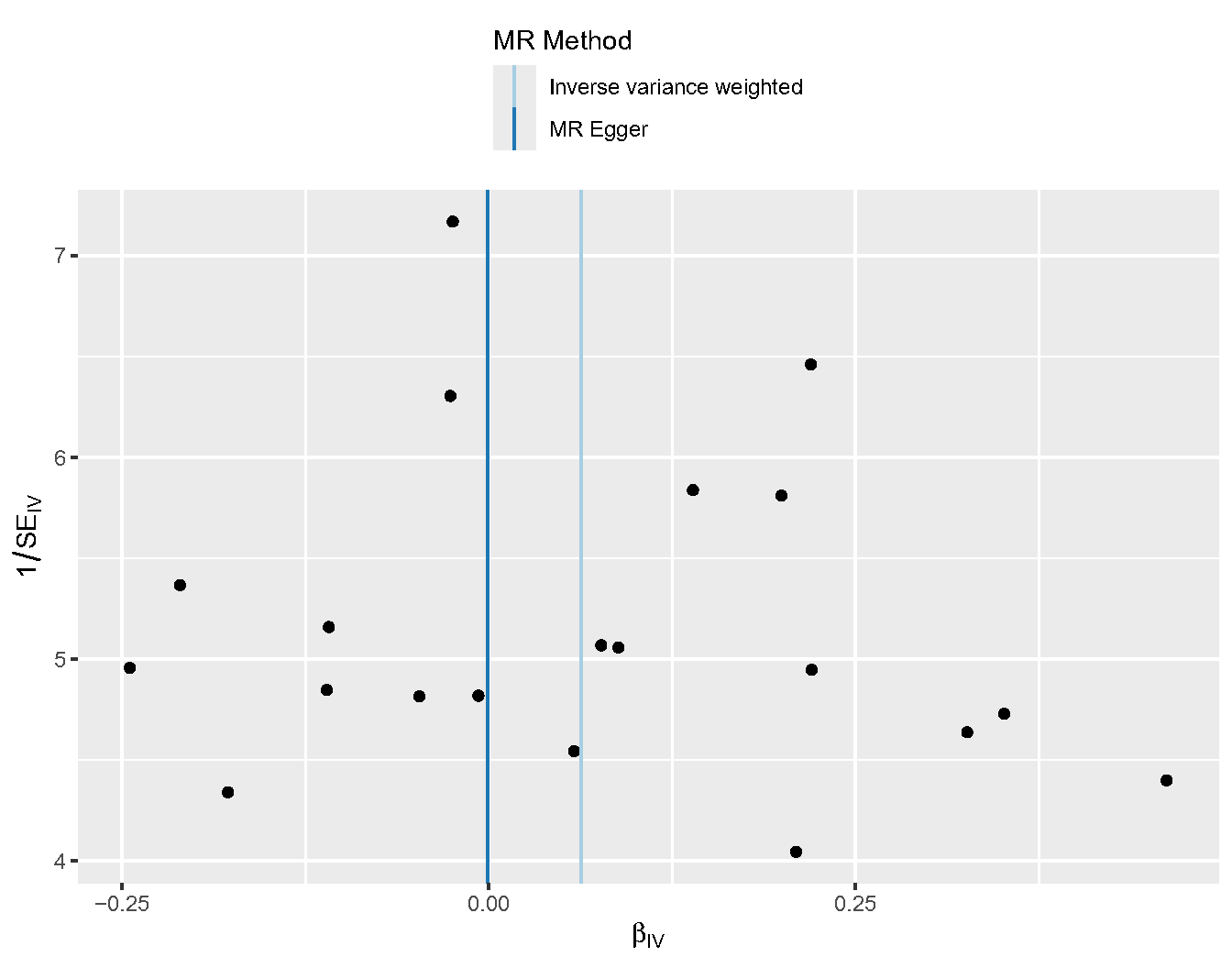


**Figure S2(D).**

Supplement: Supplementary file 9 [file medi-105-e48963-s009.docx]

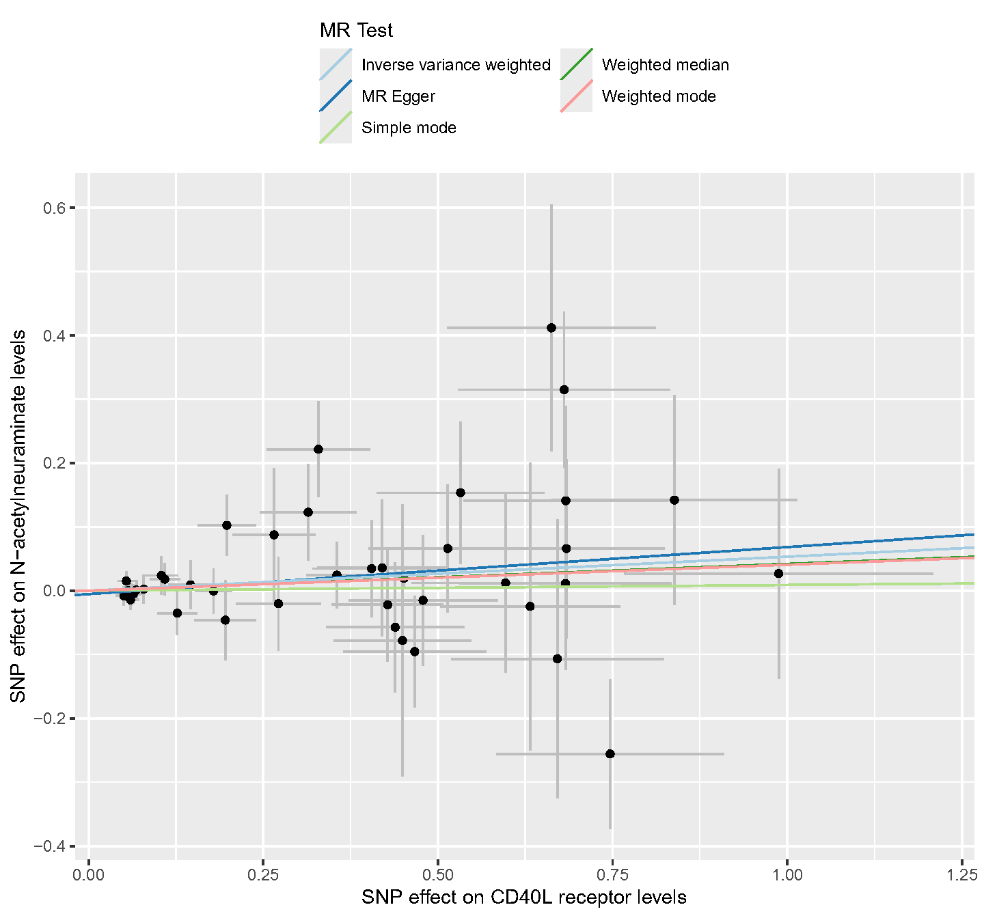


**Figure S3(A).**


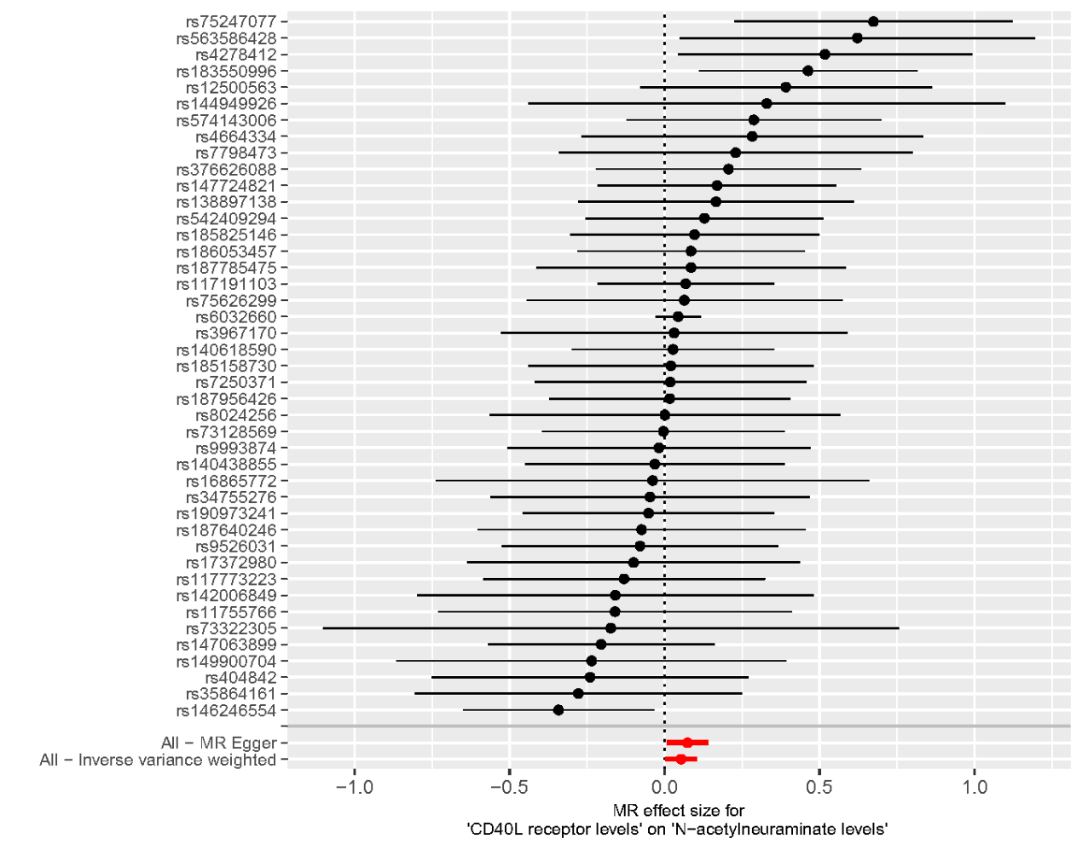


**Figure S3(B).**


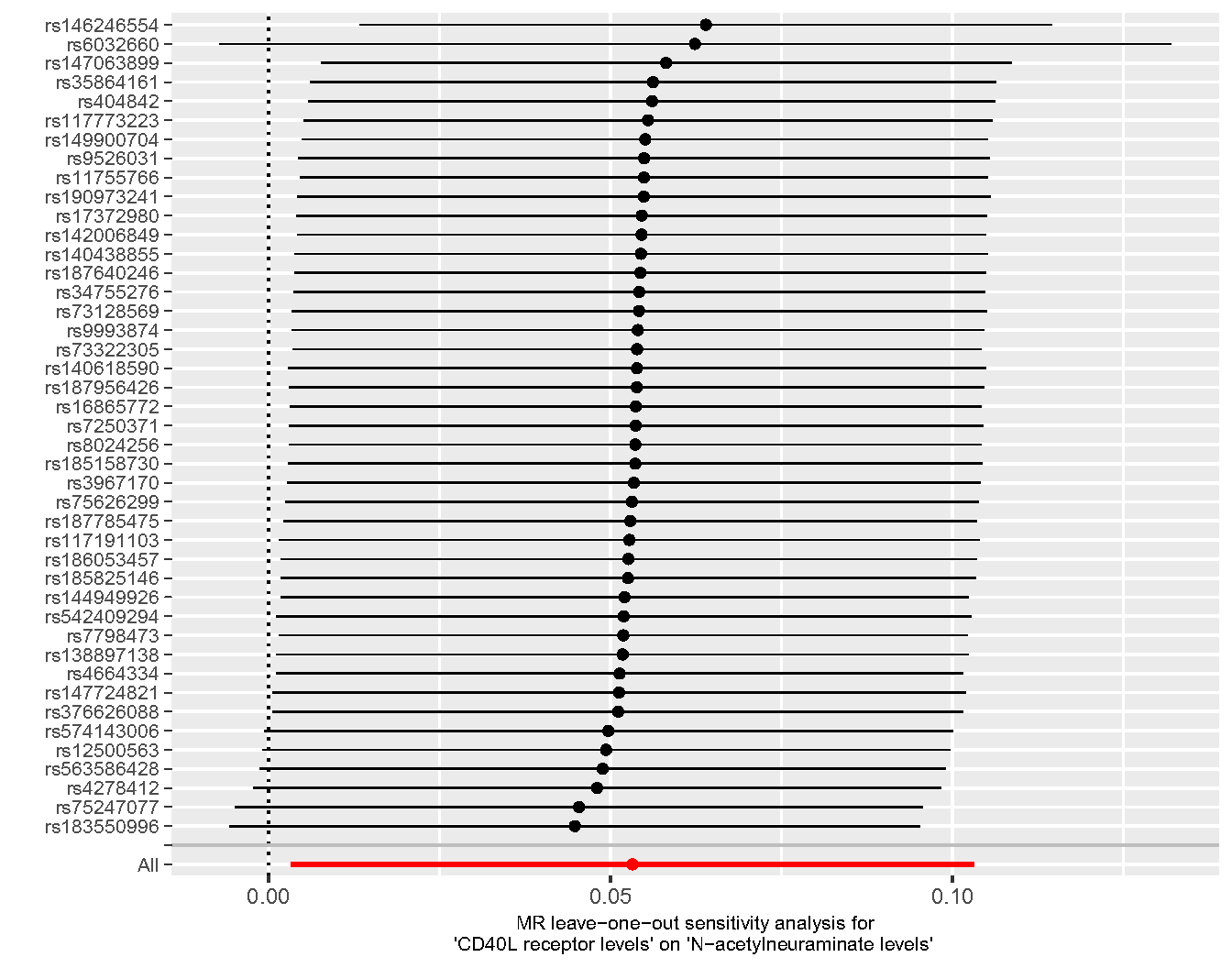


**Figure S3(C).**


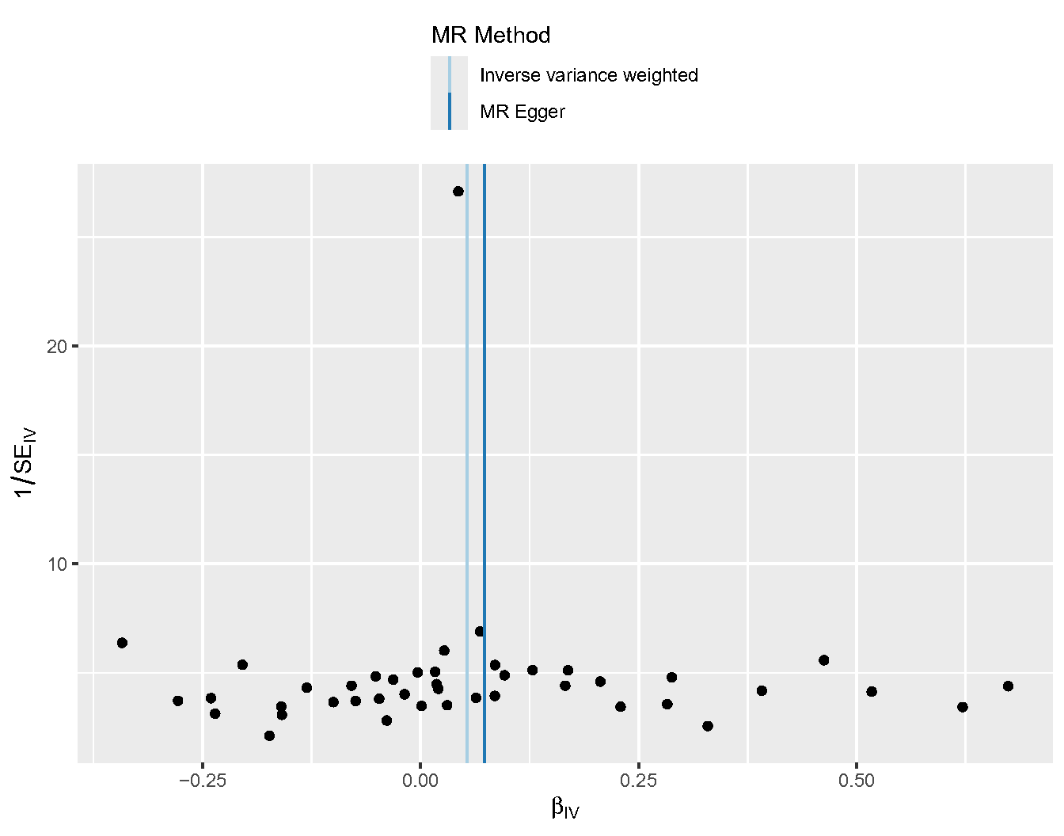


**Figure S3(D).**

Supplement: Supplementary file 11 [file medi-105-e48963-s011.docx]

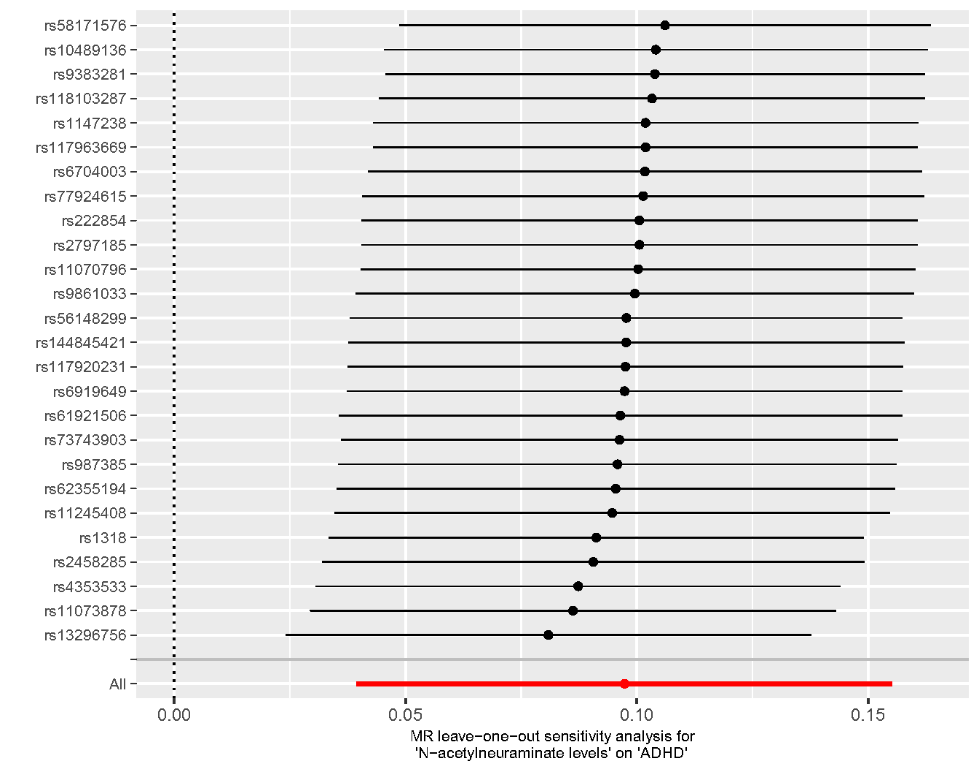


**Figure S4(A).**


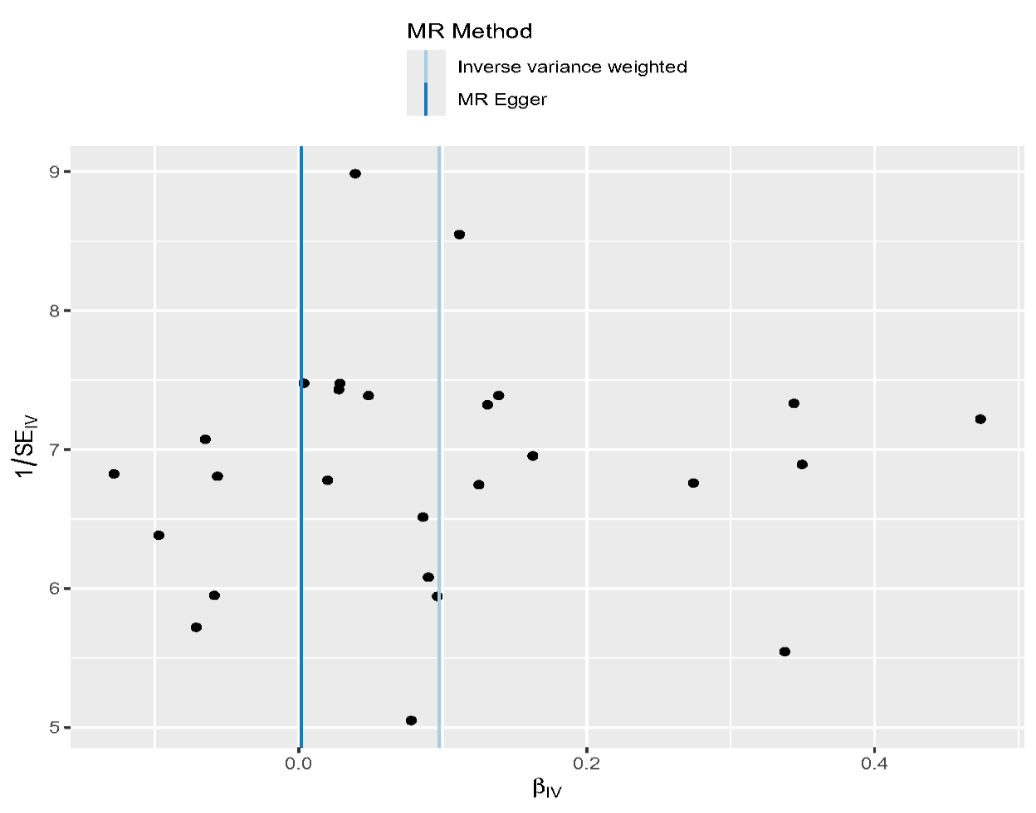


**Figure S4(B).**

Supplement: Supplementary file 18 [file medi-105-e48963-s018.docx]
